# Systematic Classification of Disease Severity for Evaluation of Expanded Carrier Screening Panels

**DOI:** 10.1371/journal.pone.0114391

**Published:** 2014-12-10

**Authors:** Gabriel A. Lazarin, Felicia Hawthorne, Nicholas S. Collins, Elizabeth A. Platt, Eric A. Evans, Imran S. Haque

**Affiliations:** Counsyl, South San Francisco, California, United States of America; Stavanger University Hospital, Norway

## Abstract

Professional guidelines dictate that disease severity is a key criterion for carrier screening. Expanded carrier screening, which tests for hundreds to thousands of mutations simultaneously, requires an objective, systematic means of describing a given disease's severity to build screening panels. We hypothesized that diseases with characteristics deemed to be of highest impact would likewise be rated as most severe, and diseases with characteristics of lower impact would be rated as less severe. We describe a pilot test of this hypothesis in which we surveyed 192 health care professionals to determine the impact of specific disease phenotypic characteristics on perceived severity, and asked the same group to rate the severity of selected inherited diseases. The results support the hypothesis: we identified four “Tiers” of disease characteristics (1–4). Based on these responses, we developed an algorithm that, based on the combination of characteristics normally seen in an affected individual, classifies the disease as Profound, Severe, Moderate, or Mild. This algorithm allows simple classification of disease severity that is replicable and not labor intensive.

## Introduction

Genetic carrier screening aids reproductive planning by identifying individuals at increased risk of bearing children with autosomal recessive diseases. Carrier screening for certain severe childhood genetic diseases, such as cystic fibrosis (OMIM #219700), sickle cell disease (OMIM #603903), and Tay-Sachs disease (OMIM #272800), has been well-established for decades. The two major medical organizations issuing recommendations on genetic carrier screening in the United States are the American College of Medical Genetics and Genomics (ACMG) and the American College of Obstetricians and Gynecologists (ACOG). Both ACOG and ACMG recommend offering carrier screening for some diseases (e.g., cystic fibrosis) to the general population, and each society recommends a different set of diseases to be additionally offered to individuals of ethnicities at higher prior risk of carrying those diseases.

Traditional methods of genetic testing, such as dot-blot hybridization, oligonucleotide ligation, and Sanger sequencing [1], have limited throughput and relatively high per-patient cost; as a consequence, genetic screening has historically been performed for only the most severe and prevalent conditions to ensure cost-effectiveness. The advent of high-throughput platforms such as microarrays [2], microfluidic genotyping [3], [4], and next-generation sequencing [5] has allowed the integration of hundreds to thousands of mutations on a single screening panel, for little additional assay cost versus a traditional screening panel. Several commercial laboratories now offer such “expanded carrier screening” (ECS). Clinical data demonstrates that most individuals are carriers of diseases not yet included in screening guidelines [6].

The ability to inexpensively integrate large numbers of diseases into screening panels necessitates a systematic effort to prioritize diseases for inclusion. Recently, ACMG issued a position statement on expanded carrier screening [7]. The guidelines specify that to be included on an ECS panel, “Disorders should be of a nature that most at-risk patients and their partners identified in the screening program would consider having a prenatal diagnosis to facilitate making decisions around reproduction,” and that “there must be validated clinical association between the mutation(s) detected and the severity of the disorder.” It is thus clear that disease severity is a critical criterion in the categorization of diseases for genetic screening.

Previously, ACMG engaged in a large study of disease characteristics to prioritize conditions for newborn screening (NBS) [8]. In this study, 292 individuals evaluated 84 conditions (with a total of 3,949 evaluations) for various properties, including disease severity. While this 241-page study was a landmark in disease evaluation, its survey and manual integration methodology is highly labor-intensive. As new diseases are mapped to specific genetic loci and technology enables testing for new conditions, it is impractical to repeatedly perform such a technique for disease ranking.

In this paper, we present a pilot test of a ranking method to semi-automatically categorize the severity of a genetic disease in the context of defining a screening panel. The model was built by surveying health care professionals (physicians and genetic counselors) simultaneously about the severity of diseases and the severity of their characteristics. We demonstrate that a simple method that considers gross clinical characteristics of a genetic disease accurately reproduces its clinical severity assessment. Therefore, our model allows rapid categorization of the severity of a disease, given knowledge of its characteristics (i.e., only a definition of the disease).

## Materials and Methods

**Figure 1:**
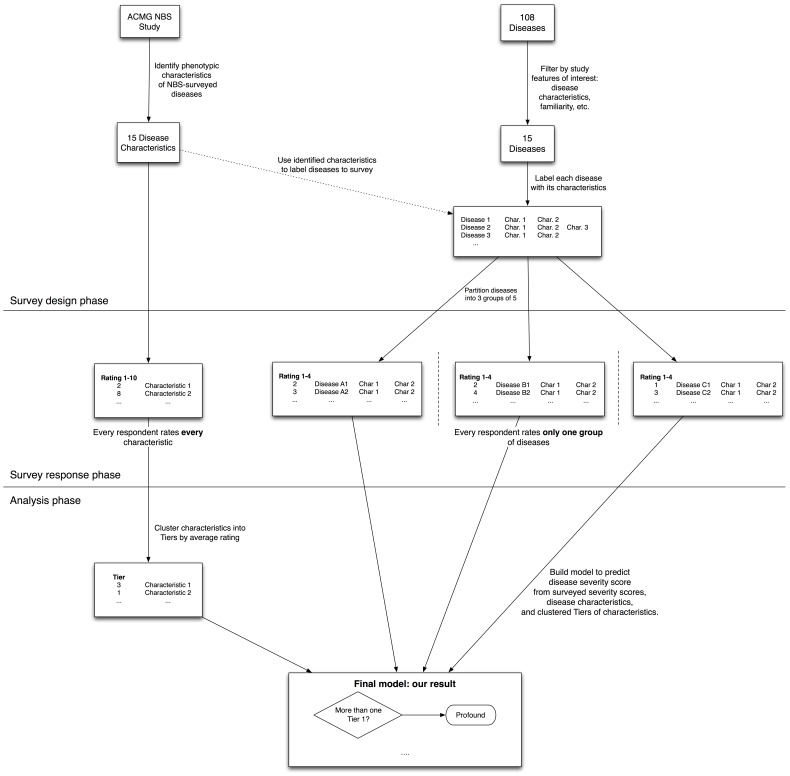
Overall study design.

Our hypothesis was that the rated severity of a disease could be expressed as a simple relation of the disease's clinical characteristics (e.g., shortened lifespan or sensory impairment) and severity ratings of the individual characteristics. If true, this hypothesis would allow a workflow in which disease characteristics may be surveyed for severity once, and the scoring system generalized to classify the severity of any disease thereafter, without requiring re-survey for each additional disease. The overall structure of our study is illustrated in Fig. 1.

To test this hypothesis, we distributed a survey to healthcare professionals who may offer carrier screening. The survey distributed to each respondent consisted of two components. The first component asked respondents to rate various disease clinical characteristics on a 1–10 scale (1 =  least important) in terms of how important they were to determine disease severity. The second component asked the respondent to rate the severity of five diseases on a scale from 1 to 4 (1 = Mild, 2 = Moderate, 3 = Severe, 4 = Profound). Respondents were provided with characteristics of each disease. Three different surveys were distributed, with each type containing a different set of five diseases. Each respondent was assigned one of the three survey versions at random.

N = 3184 physicians, genetic counselors, and geneticists with current email addresses from our internal database were invited to participate in the online survey. The survey was available for two weeks in April 2013. Invitation to participate was not dependent on the use of our laboratory for clinical testing; providers in our database had previously volunteered their contact information. A single raffle prize (iPad, Apple Corporation, Cupertino, CA) was offered for interested survey participants. Survey respondents were identified by email address only for the purposes of the random raffle; all analysis was performed on anonymized data lacking personal identifiers. The invitation email indicated the voluntary nature of this survey and identified our study's objective: to describe how disease severity might be categorized.

### Survey of Disease Characteristics

We identified thirteen common characteristics of inherited disorders, identified via review of characteristics of diseases in the ACMG NBS survey [8]. We further included two “modifier” properties, which are not characteristics of disease progression *per se*, that we thought might affect severity rankings of diseases: variable expressivity and availability of treatment. Despite the exclusion of “availability of treatment” from the severity criteria in the ACMG NBS survey [8], we included it in our survey because that survey included availability of treatment in their considerations of whether or not to screen for a disease. All characteristics surveyed can be seen in Table 1.

**Table 1:** Ratings of disease characteristics.

Disease Characteristic	Avg	Median	Min	Max
**Tier 1**				
Shortened life span: infancy	9.53	10	1	10
Shortened life span: childhood/adolescence	9.17	9	2	10
Intellectual disability	9.07	9	5	10
**Tier 2**				
Shortened life span: premature adulthood	8.01	8	1	10
Impaired mobility	7.98	8	2	10
Internal physical malformation	7.71	8	3	10
**Tier 3**				
Sensory impairment: vision	6.98	7	1	10
Immunodeficiency/cancer	6.76	7	1	10
Sensory impairment: hearing	6.67	7	1	10
Sensory impairment: touch, other (including pain)	6.65	7	1	10
Mental illness	6.54	7	1	10
Dysmorphic features	6.43	7	1	10
**Tier 4**				
Reduced fertility	3.97	3	1	10
**Severity Modifiers**				
Availability of treatment	8.07	9	1	10
Variable expressivity	6.14	6	1	10

**Figure 2:**
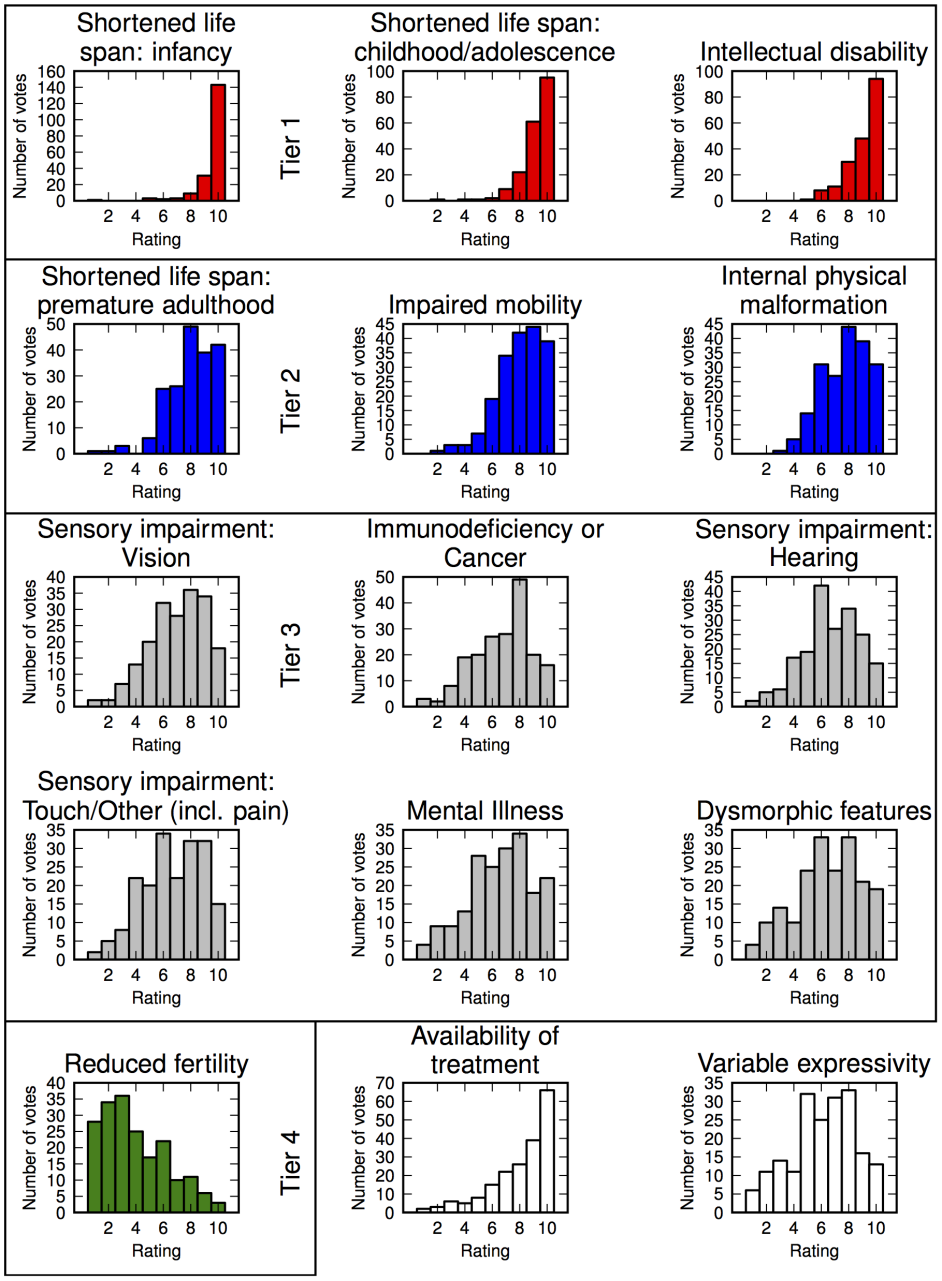
Distribution of disease characteristic severity ratings.

Tiers determined by hierarchical clustering as described in Methods. Full distributions are shown for each in Fig. 2.

The first survey component, distributed identically to all survey participants, asked the respondent to rate between 1 and 10 the importance of all 15 disease characteristics when determining the severity or burden of a disease (1 = least important).

Respondents were asked to quantify the importance of reduced penetrance or variable expressivity in two ways. First, it was included as a characteristic to be ranked from 1 to 10. In a separate question, respondents were asked what percentage of affected individuals should present a characteristic for it to be taken into account when assessing disease severity (>25%, >50%, >75%, 100%).

### Disease-specific Severity Ratings

As the pilot test of this method, we selected a subset of fifteen diseases from the 108-disease panel offered by Counsyl, a laboratory offering expanded carrier screening. We aimed to create three sets of five diseases, each with a balanced representation of several disease features: recognizable versus unfamiliar diseases (e.g., cystic fibrosis versus homocystinuria (OMIM# 236200); many versus few severity features (e.g., Smith-Lemli-Opitz syndrome (OMIM# 270400) versus *GJB2*-related deafness (OMIM# 220290)); and hypothesized high severity versus hypothesized low severity (e.g., fragile X syndrome (OMIM# 300624) versus alpha-1 antitrypsin deficiency (OMIM# 613490)). We also tried to balance the number of diseases included in previous screening recommendations. Most (12/15) of the diseases we surveyed were also surveyed in the ACMG report on newborn screening [8]. Of these twelve, half were recommended for newborn screening. Three of the surveyed diseases are currently recommended to be offered for universal or ethnicity-specific screening by ACMG or ACOG. A table of diseases and their screening status is given in Table 2.

**Table 2:** Number of respondents for each set of diseases and disease features.

Survey Set	Respondents, n	Disease	ACMG/ACOG	Assessed for NBS	Included in NBS
Set A	52	beta-thalassemia/sickle cell disease	X	X	X
		citrullinemia type 1		X	X
		GJB2-related nonsyndromic hearing loss and deafness		X	X
		short chain acyl-CoA dehydrogenase deficiency		X	
		Usher syndrome type 1F			
Set B	46	Bardet-Biedl syndrome			
		cystic fibrosis	X	X	X
		fragile X syndrome		X	
		homocystinuria caused by cystathione beta-synthase deficiency		X	X
		Smith-Lemli-Opitz syndrome		X	
Set C	94	alpha-1 antitrypsin deficiency		X	
		Canavan disease	X		
		Galactosemia		X	X
		Pompe disease		X	
		Wilson disease		X	

These diseases were then divided into three sets of five, each designed according to the criteria for disease selection above (e.g., a mix of familiar and unfamiliar diseases on each set). For the second component of the survey, each respondent was assigned one of these sets at random. Respondents were asked to consider the natural, untreated course of each disease and to rank its severity on an ordinal scale of Mild, Moderate, Severe, or Profound. Both the emphasis on untreated disease courses and the ordinal scale followed the methodology of the ACMG 2006 NBS survey [8]. For each disease, a table was provided indicating which of the fifteen characteristics from the first section applied to the natural progression of the condition. The list of characteristics assigned to each disease was based on a reading of disease natural histories through sources such as GeneReviews [9], and literature cited within. They are listed in Table 3.

**Table 3:** Severity ratings and disease characteristics for each disease surveyed.

Group	Disease	Average	Median	Min	Max
**Profound**	Canavan disease: *LE* a *: childhood/adolescence; intellectual disability; impaired mobility; vision impairment; hearing impairment; sensory impairment: other; dysmorphic features*	3.66	4	2	4
	Smith-Lemli-Opitz syndrome: *LE: infancy; intellectual disability; impaired mobility; internal physical malformation; mental illness; dysmorphic features*	3.54	4	2	4
	Citrullinemia type 1: *LE: infancy; intellectual disability; impaired mobility; internal physical malformation; vision impairment; sensory impairment: other*	3.54	4	2	4
	Pompe disease: *LE: childhood/adolescence; intellectual disability; impaired mobility; internal physical malformation; immunodeficiency/cancer; dysmorphic features*	3.48	4	2	4
	Galactosemia: *LE: infancy; intellectual disability; impaired mobility; vision impairment; immunodeficiency/cancer; mental illness*	3.46	4	2	4
**Severe**	Homocystinuria caused by CBS deficiency: *Intellectual disability; LE: premature adulthood; impaired mobility; internal physical malformation; vision impairment; sensory impairment: other; mental illness; dysmorphic features*	3.13	3	2	4
	Cystic fibrosis: *LE: childhood/adolescence; impaired mobility; internal physical malformation; immunodeficiency/cancer*	2.98	3	2	4
	Short-chain acyl-CoA dehydrogenase deficiency: *LE: childhood/adolescence; impaired mobility; mental illness; dysmorphic features*	2.92	3	1	4
	Wilson disease: *Intellectual disability; LE: premature adulthood; impaired mobility; internal physical malformation; mental illness*	2.86	3	2	4
	Bardet-Biedl syndrome: *Intellectual disability; internal physical malformation; vision impairment; mental illness; dysmorphic features*	2.83	3	1	4
	Fragile X syndrome: *Intellectual disability; impaired mobility; vision impairment; immunodeficiency/cancer; mental illness; dysmorphic features*	2.83	3	1	4
	Beta-thalassemia/sickle cell disease: *LE: premature adulthood; impaired mobility; internal physical malformation; immunodeficiency/cancer; sensory impairment: other*	2.79	3	2	4
	Usher syndrome type 1F: *Impaired mobility; vision impairment; hearing impairmen; sensory impairment: other*	2.65	3	1	4
**Moderate**	GJB2-related nonsyndromic hearing loss and deafness: *Hearing impairment*	1.85	2	1	4
	Alpha-1 antitrypsin deficiency: *Impaired mobility; internal physical malformation; immunodeficiency/cancer*	1.84	2	1	4

**Figure 3:**
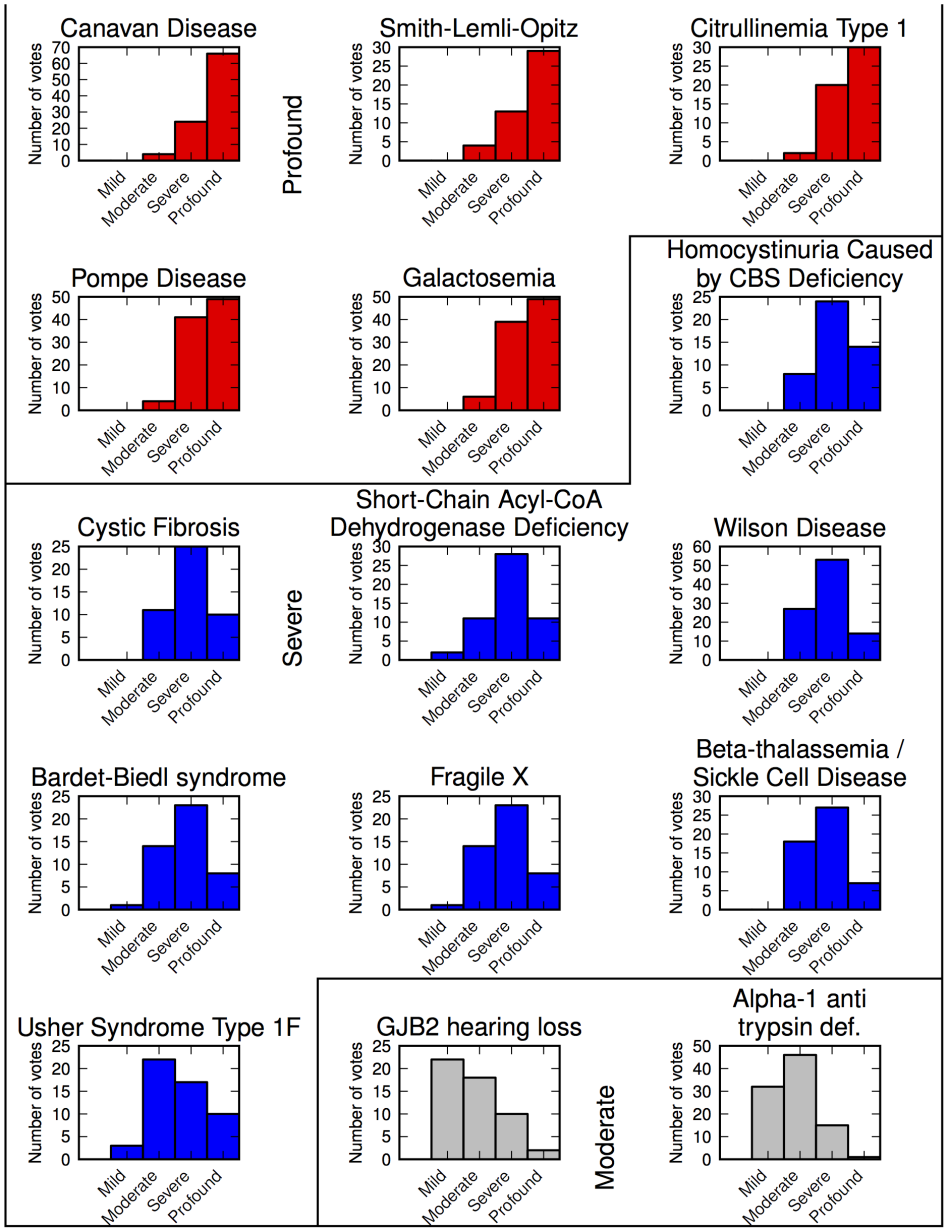
Distribution of disease severity ratings.

Clusters determined by hierarchical clustering as described in Methods. Full distributions are shown in Fig. 3. Disease characteristics are described and ordered according to highest impact, as assessed in Table 2.

a :LE  =  life expectancy.

### Analysis

The importance values (1–10) of each disease characteristic were averaged across all respondents. Following the ACMG NBS report methodology, we similarly converted the disease severity rankings to numbers 1–4 (1 = Mild, 4 = Profound), and averaged them across respondents. Ward's hierarchical clustering [10] as implemented in scikit-learn [11] was used to group the surveyed diseases and characteristics on the basis of their scores. Ward's method is a hierarchical clustering technique which, at each step, chooses to merge the two clusters that will produce the smallest increase in intra-cluster variance.

## Results

### Survey Respondents

Of 3185 invited healthcare professionals, n = 192 (∼6.4%) completed the survey. 70.3% of respondents indicated that genetic counseling was their primary profession; almost all of the rest (29.2%) indicated that they were physicians. 31.3% of respondents held a doctoral degree (MD/DO/PhD). Complete demographics of the survey respondents are listed in Table 4.

**Table 4:** Demographics of survey respondents.

General	
Number of Respondents	192
Average time to completion^a^	5 min, 42 sec
**Profession**	**n**
Genetic Counselor	70.3%
Physician	29.2%
Other Health Care Professional	1%
**Degree** b	**n**
MD/DO	29.2%
PhD	2.1%
MS	68.2%
Other	0.5%
**Specialty** b	**n**
OB/GYN	33.9%
Perinatology	24.5%
IVF	11.5%
Genetics	58.9%
Pediatrics	10.4%
Other	9.9%

a Excludes three surveys which took > 3 hours to complete.

b Respondents were asked to mark all that apply.

### Disease Characteristics Ratings

Ratings of the 15 disease characteristics fell into four clusters (“Tiers”), with average rankings>9 (Tier 1), around 8 (Tier 2), between 6 and 7 (Tier 3), and below 6 (Tier 4). The average rating for each characteristic is presented in Table 1, sorted by severity ranking. The full distribution is presented in Fig. 2.

The three characteristics with highest perceived severity (Tier 1) coincided with the most severe set found in the NBS criteria: infant life span (average rating 9.53), childhood/adolescent life span (9.17), and intellectual disability (9.07). The next-highest-ranked group of characteristics included shortened adult life span (8.01), impaired mobility (7.98), and internal physical malformation (7.71). Tier 3 included vision impairment (6.98), hearing impairment (6.67), touch or other sensory impairment (including persistent pain, or pain insensitivity) (6.65), immunodeficiency or cancer (6.76), mental illness (6.54), and dysmorphic features (6.43). Tier 4 consisted of only reduced fertility, which was a clear outlier from the other characteristics (average rating 3.97).

Practitioners taking the survey regarded variable expressivity as being a factor of moderate importance when determining disease severity (average rating 6.14, at the bottom of Tier 3). They agreed that availability of treatment is quite significant to determining severity: availability of treatment had an average rating of 8.07, at the top of Tier 2. While practitioners agreed that variable expressivity is important, there was little consensus on the quantitative penetrance required for a feature to be considered in severity estimation – n = 17 (8.9%) responded>0%, n = 75 (31.9%) responded>25%, n = 63 (32.8%) responded>50%, n = 36 (18.8%) responded>75%, and n = 1 (0.5%) responded 100%. The mode of the distribution lay between “>25%” and “>50%” penetrance required.

### Disease-specific severity ratings

Healthcare professionals each evaluated one randomly-assigned set of five diseases for severity (15 diseases assessed in total), on an ordinal scale of “Mild”, “Moderate”, “Severe”, or “Profound”; ordinal ranking were converted to integers 1–4 for analysis. Disease subsets and response counts are shown in Table 2. One subset (C) had nearly twice the number of responses (set C n = 94; set A n = 52; set B n = 46) as the other two. The reason for such divergence is unclear, but no obvious statistical divergence was seen in the ratings as a consequence.

No surveyed diseases had an average rating below 1.8 of 4; thus, no diseases were classified as Mild. Diseases were assigned severity classes by three-class hierarchical clustering for Profound, Severe, and Moderate. Diseases, their average severity ratings, and assigned severity clusters are shown in Table 3. The full ratings distribution is presented in Fig. 3.

## Discussion

### Data Quality and Biases

The survey data reported here represent a sizable effort to evaluate the opinions of healthcare professionals regarding the severity of genetic disease. The sample size (N = 192 respondents) is similar in magnitude to that reported for the ACMG NBS guidelines (N = 292). Survey participants included both genetic counselors as well as physicians specializing in genetics and/or reproductive care. This representation from both key medical parties in preconception/prenatal screening lends credibility to the accuracy of representation of medical opinion. A potential limitation is that participants may not have expert knowledge in the diseases they assessed. Focusing a similar study on such an expert population is of interest in determining reproducibility. However, it is also likely that these providers are routinely discussing carrier screening with the general population (in particular, genetic counselors may be particularly suited for describing disease severity). We propose the data still have merit independent of disease expert inclusion.

While the survey was authored and distributed by a laboratory specializing in genetic screening, to avoid bias it was sent out to an existing list of healthcare professionals regardless of whether they sent patient specimens to the laboratory. Since their contact information was provided to us voluntarily, there may be potential bias toward those with greater carrier screening knowledge or interest.

Our survey respondents were healthcare providers who are familiar with traditional and expanded carrier screening. While this population has obvious relevant perspective, it may differ from that of reproductive-age individuals. In fact, these differences have been previously observed [12]. A prudent corollary study would survey patients for comparison.

### Disease severity corresponds with disease characteristics

The study's main objective was to determine whether disease-specific surveys to determine severity could be replaced by a simpler procedure focusing only on disease characteristics and the severity of those characteristics.

Table 3 shows the surveyed diseases, grouped by severity tier, and which characteristics each disease exhibits in its untreated form. The most-severe (Profound severity) cluster of diseases included, for example, Canavan disease and Smith-Lemli-Opitz syndrome. Diseases in this group, when untreated, all have a risk of intellectual disability in addition to a reduction of lifespan to infancy, childhood, or adolescence. Diseases outside this tier typically do not exhibit both characteristics. The next group of diseases (Severe) included, for example, cystic fibrosis, fragile X syndrome and Usher syndrome type 1F. All but one of the Severe-tier diseases, when untreated, have either a risk of intellectual disability or a shortened life expectancy to infancy, childhood, or adolescence (Usher syndrome type 1F is the outlier in the group). The Moderate tier, including GJB2-related hearing loss and alpha-1 antitrypsin deficiency, did not include any diseases affecting intellectual disability or life expectancy; neither did these diseases result in the loss of more than one sense, nor the loss of a single sense and a loss in mobility.

### Proposed severity classification criteria

Our data corroborate our hypothesis that characteristics rated in higher importance tiers align with diseases rated in higher severity tiers. The two clusterings were done independently on their own importance ratings (i.e., characteristic ratings in Table 1 did not affect the disease ratings in Table 3, and vice versa). In particular, Tier 1 characteristics appear to be the most significant distinguishing factor between disease severity groupings.

Three characteristics were eliminated from the ranking scale because they were found not to affect disease severity scores: availability of treatment, variable expressivity, and reduced fertility. Reduced fertility was a clear outlier among the surveyed criteria, with a much lower score than any other characteristic. Availability of treatment is not a measure of the severity of an untreated disease. However, it was rated as highly important (more so than any sensory deficit); thus, while it is not sensible to include it in an assessment of untreated severity, it is reasonable to consider it in conjunction with severity when considering disease inclusion criteria. Unfortunately, the survey's design makes it difficult to interpret responses to this characteristic: it is not clear whether respondents believed that the *presence* or *absence* of treatment was of importance. Finally, we captured variable expressivity in the more detailed question on disease penetrance. A plurality of respondents to this question stated that a characteristic would be important to the severity of a disease if at least 25% of patients exhibited it.

**Figure 4:**
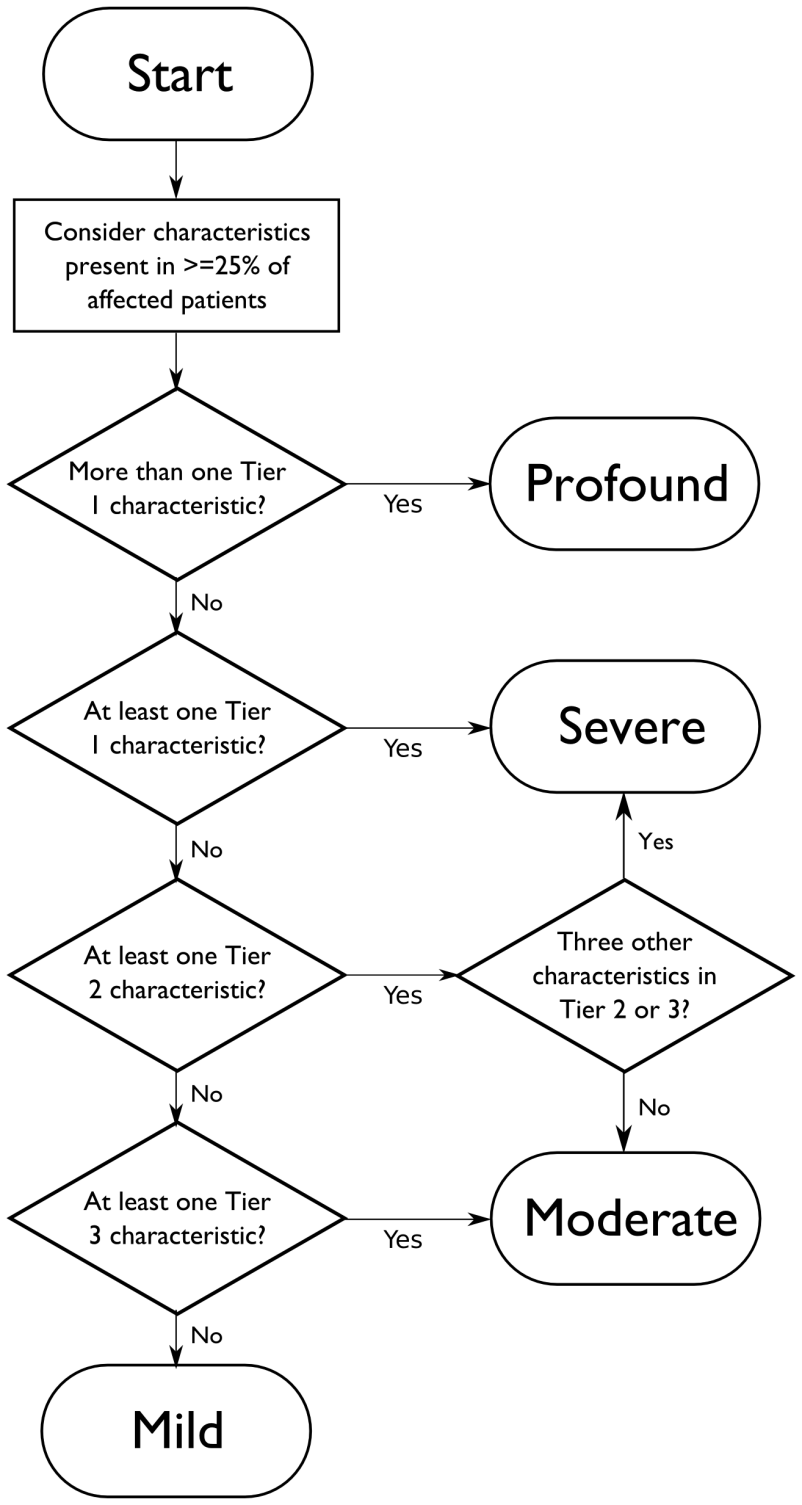
Proposed decision algorithm for severity classification.

We propose a preliminary set of criteria for disease severity classification, summarized in Fig. 4. A characteristic should be considered in a disease's severity ranking if at least 25% of affected individuals show the characteristic. Diseases are classified as Profound if their untreated course includes more than one Tier 1 characteristic. Diseases may be classified as Severe on either of two paths: first, if they exhibit a single Tier 1 characteristic (e.g., homocystinuria); or second, if they exhibit a Tier 2 characteristic and at least three other Tier 2 or 3 characteristics. Moderate diseases are those that exhibit at least one Tier 2 or 3 characteristic, but fail to fall meet the guidelines for Severe. Finally, Mild diseases are defined as all others that don't meet the above criteria.

Prudent application of these criteria in the clinic would require that our study be replicated to verify the association we found between characteristic-severity and disease-severity. As an initial exercise to verify the plausibility of these criteria, we applied this classification method to 12 diseases currently included in pan-ethnic or ethnic-specific guidelines by ACMG or ACOG, several of which were not included in our original survey. Of these, four may be called Profound – alpha-thalassemia (OMIM# 604131), Canavan disease, Niemann-Pick disease type A (OMIM# 257200), and Tay-Sachs disease (OMIM# 272800). The remainder are Severe.

We made two noteworthy observations during this exercise. Alpha-thalassemia, specifically Hb Barts disease, invariably causes fetal or neonatal death. While this would be presumed to be a Profound condition, it does not meet the criteria above due to lack of observable intellectual disability. This may suggest the Profound category criteria to be too strict. Likewise, familial dysautonomia carries a non-negligible risk of intellectual disability. But because a minimal 25% risk is proposed for any characteristic, the disease is also categorized as Severe, rather than Profound.

### Conclusions

To date, disease severities have been assessed on a per-disease basis, often by polling as in the ACMG report on newborn screening [8]. To our knowledge, this paper is the first attempt to correlate general disease characteristics with severity level on a multiple-disease scale to allow systematic classification of disease severity.

We find that classifying disease severity based on a severity ranking of disease characteristics can accurately reproduce severity ratings based on per-disease polling. The disease characteristic rankings correlated with those shared in common within disease severity groups, indicating that the classification model is consistent with current perceptions of genetic disease. Those characteristics rated as more important were also defining characteristics of the more severe diseases. The two key traits identified as major predictors of disease severity – reduced life expectancy and intellectual disability – are consistent with the characteristic ranking in our survey, as well as the classifications in the ACMG NBS report [8], which is the only existing comparable guideline.

Differences in severity rankings of diseases with similar characteristics provide insight into the threshold between Severe and Moderate diseases. (For example, while both GJB2-related hearing loss and Usher syndrome type 1F are sense impairment disorders, the latter was rated as more severe presumably because it involves the loss of multiple senses.) The spectrum of rankings among diseases with severe characteristics reflects the diversity in disease severity even within these broad classifications. Thus, the classification criteria in Fig. 1 are meant to broadly categorize diseases into severity classes while simultaneously defining the criteria used to classify them.

Before incorporating this model into clinical practice, repeat studies incorporating patient and expert provider ratings, as well as additional diseases, are suitable next steps. If categorization discrepancies were to be found, there would need to be a consensus developed on the prevailing opinions – those of patients, providers that routinely discuss carrier screening, and disease experts.

Systematization of severity grading maintains transparency of process. Such transparency has numerous benefits for medical care. For example, it would aid implementation of generic informed consent as suggested by the ACMG recommendations on expanded screening [7]: a provider can communicate easily what fraction of diseases on a panel belong to each severity bin, and the particular reasons why each disease was in each bin. Systematic classification improves transparency, and by doing so improves the patient consent process, following the principles of patient autonomy and nonmaleficence.

Systematic classification of disease severity can benefit healthcare providers beyond patient consent as well. Most obviously, patient education can benefit from rankings that can be clearly tied to consensus-driven classifications (without the labor required for per-disease surveys). Laboratories can use these rankings to design multi-gene panels and convey clinical utility in an objective manner. Similarly, physicians can use these criteria to customize screening panels based on desired coverage of disease severity, even if they are not themselves expert in all the diseases available for testing.
